# Combined partial posterior fundoplication with laparoscopic sleeve gastrectomy for morbid obese patients with symptomatic GERD. Video case report

**DOI:** 10.1016/j.ijscr.2020.04.016

**Published:** 2020-05-08

**Authors:** Ozan Şen, Ahmet Gökhan Türkçapar

**Affiliations:** a Türkçapar Bariatrics, Obesity Center, İstanbul, Turkey; b Nişantaşı Üniversity, Departmant of Health Sciences, İstanbul, Turkey

**Keywords:** Bariatric surgery, Sleeve gastrectomy, Gastroesophageal reflux disease, Modified technique

## Abstract

**İntroduction:**

Gastroesophageal reflux disease (GERD), which can be seen in up to 30% in postoperative series, is perhaps the most important complication of sleeve gastrectomy(SG). The general trend for patients who are planning to have bariatric surgery and have symptomatic GERD, Roux-en-Y gastric bypass is the most common choice.

**Case Presentation and Management:**

A 42-year-old female patient with a body mass index of 36 kg/m^2^ presented to our clinic with obesity and symptomatic GERD. She had been using proton pump inhibitör (PPI) regularly for 1 year. Preoperative endoscopy showed hiatal hernia but no esophagitis. The patient underwent ambulatory pH study and GERD was confirmed. The patient was scheduled to have laparoscopic hiatal hernia repair plus combined partial posterior fundoplication and sleeve gastrectomy. Hiatal hernia was repaired, gastric fundus was passed behind the esophagus and partial posterior fundoplication was performed, and than SG was completed.

She stopped using PPI in the early postoperative period and her reflux symptoms disappeared completely. The patient lost 20 kg in the 3rd month (%40 ewl) and underwent controlled ambulatory pH moniterization and no reflux was detected.

**Conclusion:**

İn some cases this technique can be proposed to obese patients with GERD as a primary treatment modality. High numbers of patients and longer follow up care are needed to assess the long term efficacy and safety of this technique.

## Highlights

• Gastro esophageal reflux disease (GERD) remains to be an important complication after sleeve gastrectomy.
• The general trend for patients who are planning to have bariatric surgery and have symptomatic GERD, Roux-en-Y gastric bypass is the most common choice.
• Combined partial posterior fundoplication with laparoscopic sleeve gastrectomy for morbid obese patients with symptomatic GERD is a feasible and effective method.
• This technique can be proposed in some case of obese patients with GERD as primery treatment modality.

## 1 Introduction

Today, bariatric surgery is the most effective method for morbid obesity [1]. Among bariatric surgical methods, sleeve gastrectomy’s popularity (SG) has increased tremendously in the last 10 years [2,3]. However, gastroesophageal reflux disease (GERD), which can be seen in up to 30% in postoperative series, is perhaps the most important complication of SG [4,5]. On the other hand, obesity is an independent risk factor for GERD [6]. Although, GERD improves in some patients due to postoperative weight loss, de-novo GERD occurs in some patients [7]. There are studies showing improvement as well as worsening of GERD symptoms. The general trend for patients who are planning to have bariatric surgery and have symptomatic GERD or esophagitis, Roux-en-Y gastric bypass is the most common choice. However, there are also studies showing increased mild acid reflux after Roux-en-Y gastric bypass [8,9]. İn addition, the general trend in patients who previously had antireflux surgery and later became obese is in favor of Roux-en-Y gastric bypass [12,13]. İn one study, we applied SG to such patients and achieved successful results [14].

This case report was reported in accordance with the SCARE criteria [11].

## 2 Case presentation and management

**Fig. 1:**
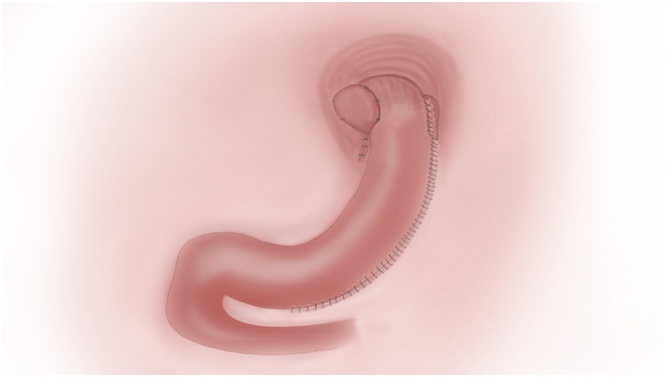
İllustration of operation technique.

A 42-year-old female patient with a body mass index of 36 kg/m^2^ presented to our clinic with obesity and symptomatic GERD. She had been using proton pump inhibitör (PPI) regularly for 1 year. Patient’s preoperative GERD-HQRL [10] (Heartburn score 0–30) was 25. The patient, also, had insulin resistance, grade 2 hepatosteatosis and dyslipidemia as comorbidities due to obesity. Preoperative endoscopy showed hiatal hernia but no esophagitis. One week after discontinuation of PPI, the patient underwent ambulatory pH study and GERD was confirmed. The patient was scheduled to have laparoscopic hiatal hernia repair plus combined partial posterior fundoplication and sleeve gastrectomy (Fig. 1). She was given thorough information about the operation and informed consent was obtained.

## 3 Surgical technique

**Fig. 2:**
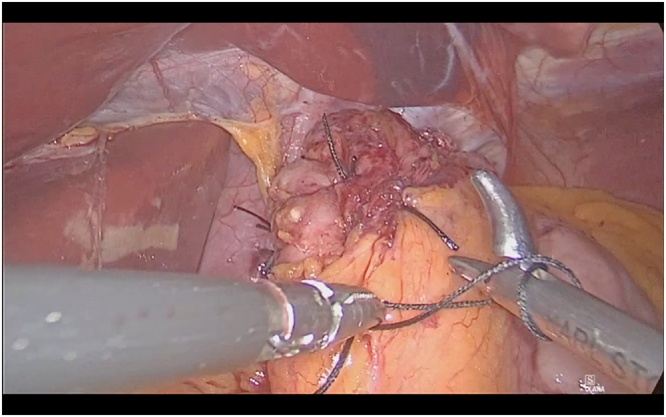
Creation of partial posterior fundoplication.

**Fig. 3:**
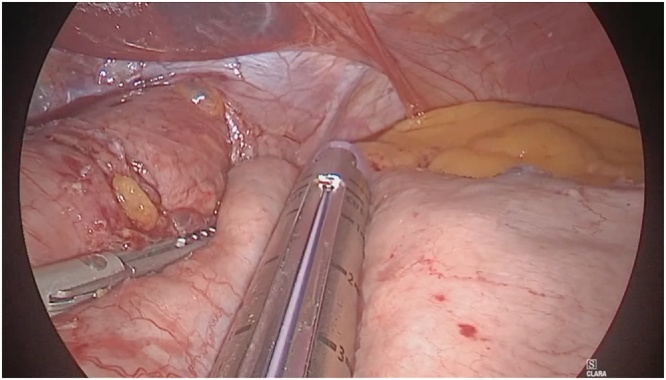
Sleeve gastrectomy was performed using Endo GIA™ 60 mm Tri-stapler.

**Fig. 4:**
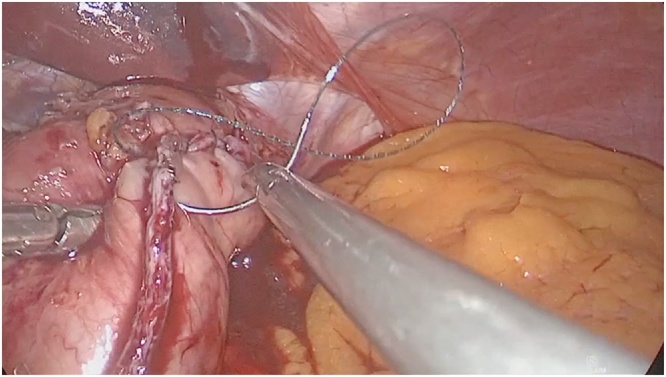
Left side of the fundoplication was created with 3.0 V-Loc and than the entire stapler line was oversewed.

The 12 mm optic trocar (Endopeth Xcel®) was entered into the abdomen under direct vision from the left supcostal area and insufflated with 12 mmHg CO_2_. SG was performed with 5 trocars. Lesser suck was opened and greator curvature was disected using harmonic scalpel. The stomach was completely released. Hiatus dissection was performed. Hiatal hernia was repaired with 2.0 etibond suture. Then, gastric fundus was passed behind the esophagus and posterior fundoplication was performed (Fig. 2). SG was completed using Endo GIA™ 60 mm Tri-stapler (4 purple and 1 brown cartridge) starting from 2 cm distance to the pylorus using 36 French bougie. After the SG was completed (Fig. 3), the stapler line was inverted at the top with 3.0 V-Loc™ and the next 3–4 cm sutures were passed through the esophagus and the left side of the fundoplication was created (Fig. 4). Then the entire stapler line was oversewed. At the last stage, gastric sleeve was fixed to the omentum at 2 points below the incisura to prevent twist. The operation took 110 min (see Video, Supplemental Digital Content, which demonstrates combined posterior fundoplication with SG).

## 4 Postoperative period

The patient recovered well and discharged home on the 3rd postoperative day. She stopped using PPI in the early postoperative period and her reflux symptoms disappeared completely. The patient lost 20 kg in the 3rd month (%40 excess weight loss) and underwent controlled ambulatory pH moniterization and no reflux was detected.

## 5 Conclusion

GERD remains to be an important problem after SG. Combined partial posterior fundoplication with laparoscopic sleeve gastrectomy for morbid obese patients with symptomatic GERD is a feasible and effective method. İn some cases this technique can be proposed to obese patients with GERD as a primary treatment modality. High numbers of patients and longer follow up are needed to assess the long term efficacy and safety of this technique.

## Funding

No funding

## Ethical approval

The patient have given her informed consent for this operation and publication.

The ethical approval has been exempted by our institution.

## Consent

The patient received a thorough explanation of this report gave her oral and written informed consent to be included in this report as well as for publication of these case, anonymous data, and pictures. A copy of the written consent is available for review on request.

## Author contribution

Each author have participated sufﬁciently in the work to take public responsibility for appropriate portions of the content. All authors met all of the following criteria:

- Substantial contributions to the conception or design of the work; or the acquisition, analysis, or interpretation of data for the work; OS and AT.

- Drafting the work or revising it critically for important intellectual content; OS.

- Final approval of the version to be published; OS.

- Agreement to be accountable for all aspects of the work in ensuring that questions related to the accuracy or integrity of any part of the work are appropriately investigated and resolved.

AT and OS operated the patient.

OS wrote the ﬁrst draft of the manuscript.

OS and AT wrote the ﬁnal draft of the manuscript.

OS made the corrections in English. All authors have reaf and approved the final report.

## Registration of research studies

Researchregistry5456.

## Guarantor

On the behalf of all author I am the guarantor

Ozan Şen

## Provenance and peer review

Not commissioned, externally peer-reviewed.

## Declaration of Competing Interest

No any conflicts of interest.
